# Retrieval-to-abstraction LLM-assisted literature synthesis: Case demonstrations in digital health technology-enabled randomized controlled trial outcome reporting

**DOI:** 10.1371/journal.pdig.0001768

**Published:** 2026-10-09

**Authors:** Taylor B. Harrison, Dian Hu, Heling Jia, Shyang Hong Tan, Fang Chen, Zhongyu Zhang, Qiuhao Lu, Jinlian Wang, Liwei Wang, Ming Huang, Larry J. Prokop, Matt Hoy, Jennifer St. Sauver, Sunyang Fu, Hongfang Liu

**Affiliations:** 1 Department of Artificial Intelligence and Informatics, Mayo Clinic, Rochester, Minnesota, United States of America; 2 Bioinformatics and Computational Biology, University of Minnesota, Minneapolis, Minnesota, United States of America; 3 McWilliams School of Biomedical Informatics, University of Texas Health Science Center, Houston, Texas, United States of America; 4 Integrated Science Program, Northwestern University, Chicago, Illinois, United States of America; 5 Department of Library Services, Mayo Clinic, Rochester, Minnesota, United States of America; 6 Division of Epidemiology, Department of Quantitative Health Sciences, Mayo Clinic, Rochester, Minnesota, United States of America; University of Chicago Pritzker School of Medicine, UNITED STATES OF AMERICA

## Abstract

Systematic reviews of randomized controlled trials (RCTs) support evidence-based clinical advancement. Clinical trial reporting guidelines, including CONSORT, establish common data elements that support systematic synthesis of published trial outcomes. However, the rapid rise of digital health technologies presents new challenges for reporting guidelines, and substantial real-world reporting heterogeneity has been observed. Therefore, machine-assisted evaluation of reporting adherence offers a pathway for more agile information management to accommodate the diverse and disruptive nature of digital health technology-enabled RCTs. Here, we propose an accelerated retrieval-to-abstraction literature synthesis framework to conduct article screening, text and table data preprocessing, and full-text review using an unmodified pretrained large language model. We apply this methodology in two digital health technology-enabled RCT case studies evaluating adherence to technology and digital health equity guideline reporting items. Large language model performance was on par with inter-annotator agreement F1 scores (≥0.9) in both screening and information-extraction tasks. These findings support the scalability and rigor of accelerated evidence synthesis and demonstrate the importance of common reporting elements across digital health technology-enabled RCTs.

## Author summary

Evidence-based medicine aligns high quality scientific evidence with real-world healthcare by integrating research findings into clinical best practices. Typically, the gold standard for updating current practice with new evidence integration is conducted through the systematic review of multiple similar clinical trials. Aggregate level insights are helpful to ensure practice changes are guided by rigorous and consistent findings. In recent years, technology has been an area of increased interest both in trial evaluation and clinical adoption to improve both patient and provider experiences. However, the rapid and diverse nature of technology innovation presents challenges for systematic evidence synthesis of similar digital health technology-enabled trials. Large language models are a type of artificial intelligence particularly well suited for organization of large scale text data, which is the main task of a systematic review and reporting guideline adherence evaluation. Therefore, we designed a retrieval-to-abstraction literature synthesis framework to help us understand current technology and digital health equity reporting practices observed in published clinical trials. This work presents an important contribution because we found that a large language model preformed as well as human annotators, while being faster and more cost-effective at the information extraction task.

## Introduction

Meta-analyses and systematic reviews of randomized controlled trials (RCTs) support evidence-based advancement of clinical practice. Reporting guidelines, such as Consolidated Standards Of Reporting Trials (CONSORT), help to establish common data elements that enable evidence synthesis across similar clinical studies [1,2]. Extensions (e.g., CONSORT-AI and CONSORT-EHEALTH) have been developed to organize the reporting of specific types of digital health technology-enabled RCTs, to include considerations such as participant computer literacy and technology stewardship (i.e., fixing bugs or content updates) [3,4]. An evaluation of 65 RCTs involving artificial intelligence (AI) interventions found that 10 of the eligible trials referenced CONSORT-AI guidelines and only 3 journals mandated its use [5]. However, despite the availability of established guidelines, it is challenging to quantify guideline adoption and substantial heterogeneity has been observed in real-world reporting practices [6,7]. Inconsistent reporting can limit the replicability and generalizability of research findings and negatively impact the quality of systematic and scoping reviews, which rely on documentation of common data elements [8].

The rapid rise of digital health technologies, some of which require evaluation through RCTs, present new barriers for existing reporting guidance and comprehensive information synthesis. Moreover, establishing aggregate-level insights about the current state of evidence through systematic review is time consuming and expensive to conduct [9,10]. Therefore, machine-assisted screening and full-text abstraction present a potential pathway to accelerate information management relating to digital health technology-enabled RCTs [11]. Natural language processing has expanded in recent years, namely, large language models (LLMs), to contribute a wide range of eligibility screening and clinical information extraction methodologies [12–15]. Furthermore, the vast training on published biomedical texts suggest opportunity to engage large language models to assist humans in systematic review tasks [16–18]. However, the promise of existing AI-assisted literature reviews have been met with concerns about trust and scalability, requiring human oversight to ensure rigor of scientific contributions [19]. To address this challenge, we designed an LLM-assisted retrieval-to-abstraction literature synthesis framework to help us understand current technology and digital health equity reporting practices observed in published clinical trials.

In this study, we present a multitask LLM-assisted evidence synthesis framework for published clinical trial outcomes. We apply our retrieval-to-abstraction process in two case studies 1) virtual reality-enabled RCTs and 2) digital health technology-enabled cognitive behavioral therapy RCTs and present solutions to unstructured text and table data parsing, followed by full-text information extraction tasks using an unmodified pretrained model. Our approach to machine-enabled information extraction with LLMs is designed as an informatics workflow to support future research questions and to accommodate custom selection of AI tools in anticipation of iterative model updates and changes in user preferences.

## Methods

We collaborated with Mayo Clinic librarians to conduct a literature search to identify published final outcomes of digital health technology-enabled RCTs using Ovid MEDLINE(R) and Epub Ahead of Print, In-Process, In-Data-Review & Other Non-Indexed Citations, Daily and Versions 1946 to February 06, 2024, that returned 16,969 results. Within this dataset, we used keyword searches to retrieve literature for two selected case studies: 1) virtual reality-enabled RCTs designed to assess model performance on one type of digital health technology reported across multiple different health specialties, and 2) digital health technology-enabled cognitive behavioral therapy RCTs designed to assess model performance on multiple technologies observed in one health specialty.

Our approach to accelerated literature synthesis leveraged a generative pre-trained transformer model, GPT-4o (OpenAI, San Francisco, California), configured for deterministic responses (standardized parameter settings: temperature 0.25, max tokens 256, top p 1, frequency penalty 0, presence penalty 0). This model was selected as a multipurpose LLM that achieved the highest performance metrics across all tasks during our previously reported evaluation of seven state-of-the-art models including GPT 3.5, GPT 4, Claude 3 Sonnet, Coral Command 0.62, Gemini 1.0 Pro, HuggingChat v0.7.0, and Llama 2 [20,21]. We addressed title screening as part of the initial Preferred Reporting Items for Systematics reviews and Meta-Analyses (PRISMA) screening phase and full-text information abstraction (Fig 1) [22]. We iteratively developed annotation guidelines and curated double-annotated gold standards for each task. We then used each task-based annotation guideline as the basis for standardized prompt development (S1 Text) and we evaluated GPT-4o performance (precision, specificity, sensitivity, negative predictive value [NPV], and F1 score) against the corresponding gold standard. All scripts are available on GitHub (https://github.com/OHNLP/ACCEL).

**Fig 1:**
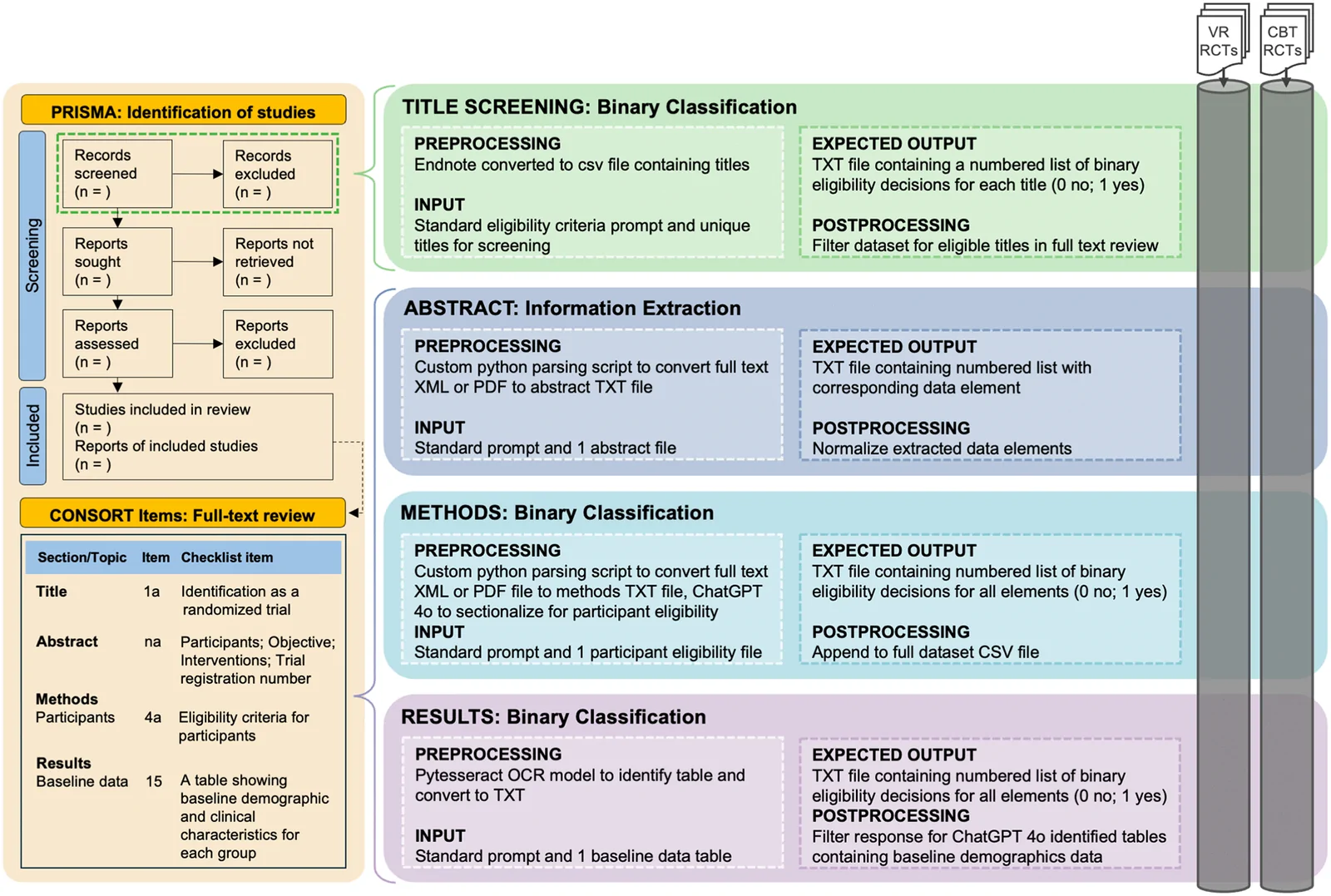
Large language model-assisted evidence synthesis workflow. Abbreviations: Preferred Reporting Items for Systematics reviews and Meta-Analyses (PRISMA), Consolidated Standards Of Reporting Trials (CONSORT), Text file (TXT), Extensible Markup Language (XML), Portable Document Format (PDF), Optical Character Recognition (OCR), Large Language Model (LLM), Inter-Annotator Agreement (IAA), F1 score (F1), and Negative Predictive Value (NPV).

### Task 1. Title screening

In accordance with CONSORT 2010 checklist item 1a, we required eligible articles to contain ‘randomized controlled trial’ or a synonym such as ‘randomised study’ within the title [1]. We conducted title screening as a binary classification task wherein the LLM determined each input to be either “eligible” or “ineligible” (S2 Text). We used in-context learning and shot-based prompts to provide the model with task definitions, as well as diverse and challenging title exemplars such as homonym terms (e.g., “tablet” meaning pill instead of a small hand-held technology) to further demonstrate the eligibility criteria (S1 Text). During our pilot testing, we identified that preservation of both performance and scalability converged when screening 25 unique titles per prompt and therefore adopted that batch size for all screening tasks [20]. We evaluated model performance favoring sensitivity to minimize this risk of potentially missing relevant articles.

### Task 2. Study abstract

We retrieved abstracts from eligible articles using the PubMed application programming interface via digital object identifier (DOI), PubMed reference number (PMID), or parsed study abstracts directly from full-text files using “PyMuPDF” and “pdfminer” Python packages. We developed and applied an annotation guideline (**S3 Text**) using MedTator (https://medtator.ohnlp.org/), a serverless open-source text annotation tool [23]. We aligned our annotation guideline with CONSORT 2010 abstract guidance, which recommends reporting details about the participants, objective, intervention, and trial registry number [24], as well as an additional item to extract information relating to the World Health Organization digital health implementations report. We developed a standardized prompt based on the manual annotation guideline for information extraction of digital tool name and trial registry number, as well as classification decisions about the digital tool user, purpose, and health specialty in which the trial was conducted (S1 Text).

1. Digital health technology name: Ontology of Digital Health Technologies developed by Hey et al [25].
2. Digital health technology user: World Health Organization digital health interventions categories for Persons, Healthcare Providers, Health Management and Support Personnel, and Data Services [26].
3. Digital health technology purpose: Our prior work identified four primary use categories of Education, Assistance, Participant, and Treatment [27].
4. Health Specialty: Association of American Medical Colleges recognized medical specialties and definitions [28].
5. Clinical Trial Registry Numbers: National and International trial registry identifiers [29,30].

### Task 3. Participant eligibility

We automatically retrieved full-text articles as extensible markup language (XML) files from PubMed, portable document format (PDF) files from EndNote 21.4, or manually through institutional library services. From full-text articles, we conducted a search for lexical variations of the word “methods” that could indicate the start of the Methods section and recorded text until encountering the end of the Methods section (e.g., a “results” header). To reduce context noise for the downstream LLM tasks, we stored the parsed Methods region for each article as a plain text (TXT) file and sectionalized the file using GPT-4o to isolate participant eligibility criteria (S1 Text). We developed an annotation guideline (S4 Text) to better understand technology related barriers for participation relating to the United States Food and Drug Administration recommendations to support digital health technologies for the advancement of trial inclusivity [31]. We used extracted information from both inclusion and exclusion criteria to identify documentation about access, usage, or requirements for the trial digital health technology, such as computer or smartphone ownership, internet and broadband access, email or website accounts, and technology literacy. We developed the standardized prompt for GPT-4o information extraction based on the annotation guideline about whether technology-related participant inclusion or exclusion criteria were reported (S1 Text).

### Task 4. Participant baseline demographic data table

We preprocessed full-text PDF files by converting each page into a portable network graphics (PNG) file using pdf2image version 1.17.0, a wrapper for pdftoppm and pdftocairo. We used Python Tesseract (Pytesseract) version 5.0.0, a Python wrapper for Google’s Tesseract-Optical Character Recognition engine, to identify table structures within each file, inclusive of multi-column, multi-page tables, and publisher style heterogeneity. Detection of tabular information triggered PNG file conversion into TXT document. We did not preprocess XML files.

We developed an annotation guideline designed to evaluate reporting of participant demographic variables for the study cohort, commonly reported in Table 1 of RCT outcome reporting [32]. We extracted information aligned with the United States Food and Drug Administration recommended reporting items and the Coss et. al. elaboration on social determinants of health including sex, race and ethnicity, education, income, employment status, housing status, disability status, insurance status, internet access, gender, and environmental safety [33,34]. We aggregated sex and gender into one category ‘sex or gender’ and race and ethnicity into one category ‘race or ethnicity’ due to ambiguous reporting practices, such as ‘female’ used in study Results to indicate both sex and gender. We applied the baseline participant demographic data table annotation guideline as a standardized prompt, wherein GPT-4o was first instructed to identify whether the TXT or XML file contained a relevant demographic table and, if so, classified each variable of interest in a binary fashion ‘reported’ or ‘not reported’ (S1 Text).

## Results

For the virtual reality case study, we retrieve 432 articles about virtual reality, augmented reality, mixed reality, simulated reality, or immersive reality. For Task 1, inter-annotator agreement for virtual reality RCT title screening was calculated from the 60% (n = 255) of titles that were double annotated resulting in an F1 score of 0.976. From the 423 total titles screened by at least one annotator, the gold standard reported 93% (n = 395) met eligibility criteria for inclusion. For the same task, the LLM achieved an F1 score of 0.968 and assigned 90% (n = 382) of titles as eligible. Of the eligible articles, we randomly selected a sample of 50 articles retrieved as full-text PDFs to evaluate preprocessing methodology across diverse publisher styles and information structures. In the second case study focused on articles using digital tools in cognitive behavioral therapy, we retrieved 164 articles about ‘cognitive behavioral therapy,’ ‘cognitive-behavioral therapy,’ ‘cognitive therapy,’ ‘behavioral therapy,’ ‘cognitive psychotherapy,’ or ‘cognitive behavioral therapy.’ We used the 60% (n = 99) of titles that were double annotated to calculate inter-annotator agreement, which resulted in an F1 score of 0.902. From the 164 total titles that were screened by at least one annotator, the gold standard reported 80% (n = 132) met eligibility criteria for inclusion, whereas the LLM achieved an F1 score of 0.939 and assigned 79% (n = 130) of titles as eligible for inclusion. From the included cognitive behavioral therapy articles, we used the PubMed Central ID Converter application programming interface to retrieve 40 studies as full-text XMLs from the PubMed Central Open Access Subset. For both case studies, we completed LLM title screening twice to assess model output reproducibility, which achieved 0.979 for the virtual reality cohort and 0.938 and for the cognitive behavioral therapy cohort. The cost to submit each text file that contained the prompt and 25 titles for evaluation did not exceed $0.02 per request.

All PDF and XML full-text files (n = 87) underwent preprocessing and information extraction of the five study abstract concepts (technology name, user, purpose, health specialty, and trial number) from Task 2, two participant eligibility criteria concepts (technology related inclusion criteria and technology related exclusion criteria) from Task 3, and 11 baseline participant demographic data table concepts (age, sex or gender, race or ethnicity, education, employment status, income, housing status, disability status, insurance status, internet access, and environmental safety) from Task 4 (Fig 2).

**Fig 2:**
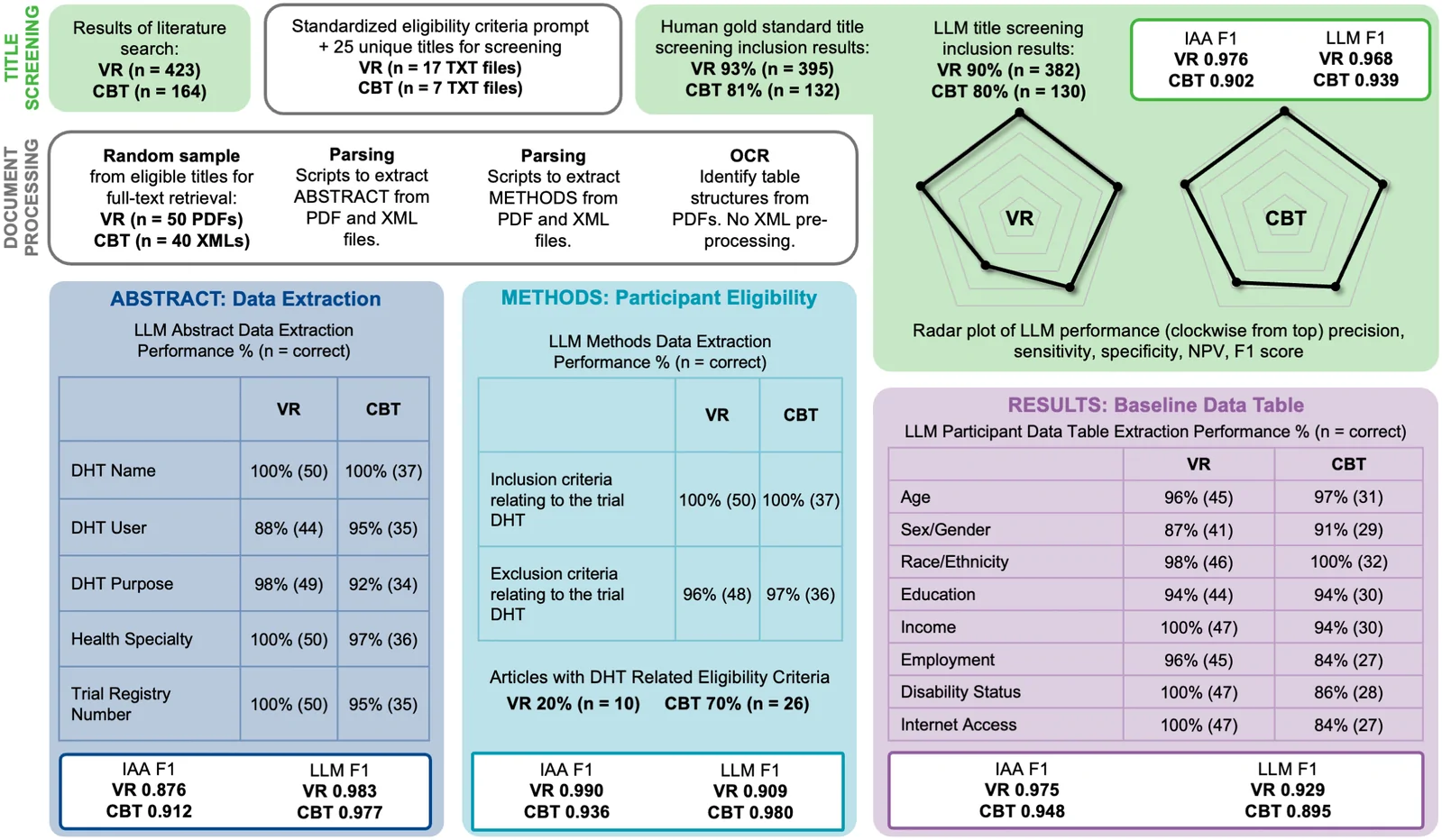
GPT-4o task performance for title screening and full-text abstraction. Abbreviations: Digital Health Technology (DHT), Virtual Reality (VR), Cognitive Behavioral Therapy (CBT), Text file (TXT), Large Language Model (LLM), Inter-Annotator Agreement (IAA), F1 score (F1) Negative Predictive Value (NPV).

In the virtual reality cohort abstract information extraction tasks, the overall inter-annotator agreement F1 score was 0.876: technology name 0.913; user 0.848; purpose 0.853; health specialty 0.870; trial number 1.000. The large language model overall F1 score was 0.968. All abstracts documented technology name, commonly listed generically as ‘virtual reality’ and a modifier such as ‘immersive,’ ‘headset,’ or ‘goggles’. Specific technology names (e.g., Oculus) were observed in eight of the trials. User was classified as ‘person’ in 35 studies, ‘provider’ in six, and both person and provider in nine trials. Virtual reality technology was used most as in the context of assistance or treatment (n = 38), such as a distraction tool to reduce anxiety and/or fear, and for education (n = 10), such as surgical training. Pediatrics (n = 7), physical medicine and rehabilitation (n = 5) and obstetrics and gynecology (n = 4) were the most common health specialties. Clinical trial numbers were reported in 11 abstracts using two nomenclature styles: National Clinical Trial number ‘NCT’ and Australian and New Zealand Clinical Trial Registry Number ‘ACTRN’. For the digital health technology-enabled cognitive behavioral therapy cohort abstract information extraction tasks the overall inter-annotator agreement F1 score was 0.912: technology name 0.873; user 0.950; purpose 0.898; health specialty 0.944; trial number 0.916. The LLM F1 score was 0.977. All abstracts reported the technology name primarily delivering cognitive behavior therapy via online/web/internet modalities (n = 35) designed for use in sub-fields of psychiatry. Users were classified as ‘Person’ in nine studies, ‘Provider’ in three, and both person and provider in 20 studies. Most cognitive behavioral therapy digital health technologies were designed for assistance or treatment (n = 25) such as therapy for drug and alcohol dependence, and for education (30%, n = 11) such as preventing suicidal ideation. The clinical trial number was reported in 27 studies beginning with the following abbreviations: Netherlands Trial Registry ‘NTR’, National Clinical Trial number ‘NCT’, Chinese Clinical Trial Registry number ‘ChiCTR’, University Hospital Medical Information Network Clinical Trials Registry ‘UMIN’, International Standard Randomised Controlled Trial Number ‘ISRCTN’, or Australian and New Zealand Clinical Trial Registry Number ‘ACTRN’.

Overall inter-annotator agreement F1 score for the virtual reality cohort participant eligibility extraction was 0.990, while the LLM achieved an F1 score of 0.909. Digital health technology-related inclusion criteria were reported in two studies and exclusion criteria were reported in 10 studies. In the digital health technology-enabled cognitive behavioral therapy cohort, the participant eligibility overall inter-annotator agreement F1 score was 0.912, whereas the LLM F1 score was 0.977. In total, 21 trials reported digital health technology-related inclusion criteria and five trials reporting technology-related exclusion criteria.

The virtual reality participant baseline demographic data table overall inter-annotator agreement F1 score was 0.975 and the LLM F1 score was 0.929. The observed reporting rate of each variable in the gold standard was as follows: Age (95%, n = 45), Sex or Gender (77%, n = 36), Education (45%, n = 21), Employment Status (23%, n = 11), Income (11%, n = 5), Housing Status (6%, n = 3), Race or Ethnicity (4%, n = 2). None of the articles reported Disability Status, Insurance Status, Internet Access, and Environmental Safety (Fig 3). In the second case study, the digital health technology-enabled cognitive behavioral therapy RCTs participant baseline demographic data table overall inter-annotator agreement F1 score was 0.948 and the LLM achieved an F1 score of 0.895. The reporting rate observed in the gold standard for each variable was as follows: Age (97%, n = 31), Sex or Gender (88%, n = 28), Education (88%, n = 28), Employment Status (66%, n = 21), Race or Ethnicity (25%, n = 8), Income (16%, n = 5), Housing Status (16%, n = 5), Disability Status (3%, 1). None of the articles reported Insurance Status, Internet Access, and Environmental Safety (Fig 3).

**Fig 3:**
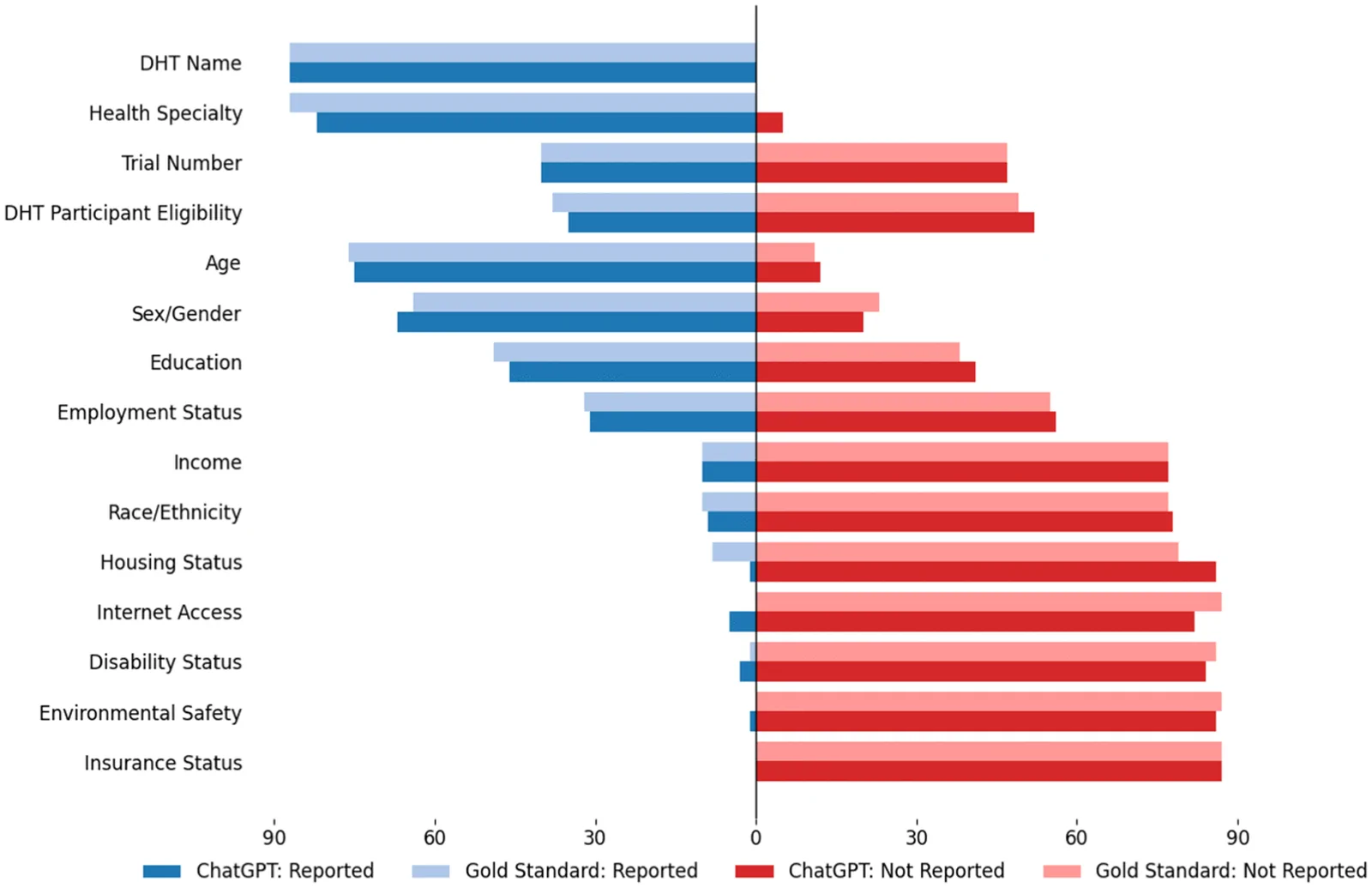
GPT-4o versus gold standard results for reported and not reported elements. The y-axis represents data elements from the information extraction and classification tasks. The x-axis represents the number of articles that reported (blue, left from center) and did not report (red, right from center) each element (n = 87 articles: virtual reality RCTs n = 50, cognitive behavioral therapy RCTs n = 37).

## Discussion

This study contributes a retrieval-to-abstraction LLM-assisted evidence synthesis framework, which we demonstrate in the context of reporting guideline adherence in virtual reality-enabled RCTs and digital health technology-enabled cognitive behavioral therapy RCTs. We leverage advancements in AI to accelerate title screening, unstructured text and table data parsing, and full-text data abstraction that rival human annotator performance across information extraction and classification tasks. We found that GPT-4o enabled screening and abstract abstraction outperformed human annotator agreement and produced similar performance across the remaining participant eligibility and participant demographic table tasks. These findings support the scalability of using LLM-assissted evidence synthesis when rigorous annotation guidelines and task-specific gold standards are curated from a sample of the data to guide prompt development and evaluate model performance. Leveraging LLMs for information synthesis of published clinical trial literature marks a period of transition from prior technology tools designed to streamline information organization for human annotators and presents opportunities to reduce time and data extraction burdens of traditional literature reviews [35,36]. As the technical capabilities of expedited, large-scale literature review advance, guideline development are adapting in parallel to ensure adequate reporting standardization, thus establishing common data elements that can be utilized for increasingly accurate LLM-enabled classification and extraction tasks. Similarly, PRISMA and PRISMA-ScR(Scoping Review extension) continue to serve as a standardization backbone with increasing importance in the era of automated information organization and synthesis.

This study has limitations, as it remains difficult to automate some aspects of the review process due to the heterogeneous nature of content, reporting structures, and publisher styling. The lack of transparency afforded by closed-source models also warrants caution and awareness for the fragility of dependence on generative AI tools. Another limitation of this approach is unknown generalizability for other topic areas and dependence on task-specific datasets developed by trained human annotators, although this workflow is intended to be less burdensome than previous completely manual processes. This framework aims to mitigate hallucinatory potential with classification-based tasks, although fine-tuning and retrieval-augmented generation may present improved performance reliability depending on the use-case domain [37–40]. By including high quality human annotation at every phase of the LLM-assisted evidence synthesis process, we provide a pathway to automation through rigorous evaluation. Furthermore, by designing a prompt engineering approach to LLM-assisted literature review using an unmodified pretrained model, we are reducing the barrier for adoption and equitable advancement of knowledge synthesis capabilities across research domains.

In conclusion, this study presents a framework toward reproducible LLM-assisted evidence synthesis to systematically understand published materials, including trends in digital health randomized controlled trials spanning technology types and health domains. We found that the LLM performed well on routine tasks such as title screening, whereas more complex tasks such as demographic table abstraction may benefit from human involvement. Our work demonstrates both the vast potential of LLM-assisted literature mining and the necessity of human evaluation at all levels of information retrieval through abstraction as a basis for which to assess task-specific model performance. Future directions to improve LLM task performance include error analysis specifically addressing negation handling, role confusion during user classification tasks, homonym language errors, as well as hallucination and sycophantic behavior.

### Data sharing

The datasets generated and analyzed during the current study as well as all code used in this manuscript are publicly available on GitHub (https://github.com/OHNLP/ACCEL).

### Ethics approval

Ethics and Consent to Participate declarations: not applicable.

## Supporting information

S1 Text Literature mining prompts used to instruct GPT-4o. Box A1. Title screening prompt - Virtual Reality Cohort Box A2. Title screening prompt - Cognitive Behavioral Therapy Cohort Box B. Abstract Information Extraction Standardized PromptBox C1. Methods Region Sectionalization for Participant Eligibility Standardized PromptBox C2. Methods Region Participant Eligibility Standardized PromptBox D1. Results Region Participant Baseline Data Table Standardized Prompt for PDFsBox D2. Results Region Participant Baseline Data Table Standardized Prompt for XMLs
(DOCX)

S2 Text Title screening eligibility criteria. (DOCX)

S3 Text Abstract annotation guidelines. (DOCX)

S4 Text Participant eligibility annotation guidelines. (DOCX)

## Abbreviation:

RCT: Randomized Controlled Trial
CONSORT: Consolidated Standards Of Reporting Trials
AI: Artificial Intelligence
LLM: Large Language Model
GPT: Generative Pre-trained Transformer
PRISMA: Preferred Reporting Items for Systematic Reviews and Meta-Analyses
NPV: Negative Predictive Value
DOI: Digital Object Identifier
PMID: PubMed reference number
XML: Extensible Markup Language
PDF: Portable Document Format
PNG: Portable Network Graphics
TXT: Plain text
