# Supplementary material for: Retrieval-to-abstraction LLM-assisted literature synthesis: Case demonstrations in digital health technology-enabled randomized controlled trial outcome reporting
Source: PLOS Digit Health. 2026 Oct 9;5(10):e0001768. doi: 10.1371/journal.pdig.0001768 (PMC13652274; doi:10.1371/journal.pdig.0001768)
Supplement: Supplementary file 1 [file pdig.0001768.s001.docx]

**SUPPLEMENTAL NOTE 1: LITERATURE MINING PROMPTS USED TO INSTRUCT GPT-4o**

Box A1. Title Screening Standardized In-Context-Learning Prompt for VR-enabled RCTs (703 tokens). Note: the response is requested as a list where the number of requested binary responses matches the number of submitted titles for evaluation. This approach was identified by the study team to mitigate hallucination evaluations exceeding the number of titles submitted.

**Objective:**

You will receive 25 article titles. For each title, determine if it is eligible based on the criteria below. Respond with only one word: "eligible" or "ineligible," as a numbered list.

**Eligibility Criteria:**

An article is considered eligible if it meets both of the following criteria:

1. The study is a randomized controlled trial (RCT). Acceptable terms include:

- Randomised controlled trial, Randomized controlled study, Randomized trial, Randomized study, Randomized control trial, Randomized control study, Randomized clinical trial, Randomized clinical study.

- Terms that describe specific types of RCT designs are also acceptable when used with RCTs: Parallel, parallel-group, non-inferiority, crossover, cross-over, factorial, pragmatic.

2. The study involves the use of virtual reality (VR). VR includes any form of immersive digital environment that uses computer-generated simulations.

**Exclusion Criteria:**

An article is ineligible if it meets any of the following conditions:

- The title includes terms like: "Proof of concept," "Feasibility," "Preliminary efficacy," "Pilot study," "Protocol," "First results," "Preliminary results," "Design," "Economic evaluation," "Health-economic evaluation," "Secondary analysis," "Qualitative study," "Follow-up," "Longitudinal study," "Short term findings," "Methods," "Letter," "Erratum," "Commentary," "Insights," "Lessons learned," "Reflections," "Correlation study," "Quasi," "Survey," "Process economic evaluation," "Bibliometric analysis," "Meta-analysis," "Systematic review," "Scoping review."

- The study does not explicitly state it is an RCT and does not involve VR.

- The title is not written in English.

**Examples of Eligible Articles:**

1. "Merged virtual reality teaching of the fundamentals of laparoscopic surgery: a randomized controlled trial."

Response: Eligible.

2. "Effectiveness of an immersive virtual reality environment on curricular training for complex cognitive skills in liver surgery: a multicentric crossover randomized trial."

Response: Eligible.

3. "Improving Cardiorespiratory and Muscular Function of Korean Firefighters: A Kinect-Based Mixed Reality Device Exercise Intervention Randomized Control Trial."

Response: Eligible.

**Examples of Ineligible Articles:**

1. "Long-term Effect of Face-to-Face vs Virtual Reality Cardiopulmonary Resuscitation (CPR) Training on Willingness to Perform CPR, Retention of Knowledge, and Dissemination of CPR Awareness: A Secondary Analysis of a Randomized Clinical Trial."

Response: Ineligible.

2. "Effects of virtual reality immersive training with computerized cognitive training on cognitive function and activities of daily living performance in patients with acute stage stroke: A preliminary randomized controlled trial."

Response: Ineligible.

3. "The Impact of Cognitive Function on Virtual Reality Intervention for Upper Extremity Rehabilitation of Patients With Subacute Stroke: Prospective Randomized Controlled Trial With 6-Month Follow-up."

Response: Ineligible.

Now, screen the following titles using the provided eligibility and exclusion criteria and respond with a numbered list selecting only "eligible" or "ineligible" for each title:

Box A2. Title Screening Standardized In-Context-Learning Prompt for DHT-enabled CBT RCTs (710 tokens). Note: the response is requested as a list where the number of requested binary responses matches the number of submitted titles for evaluation. This approach was identified by the study team to mitigate hallucination evaluations exceeding the number of titles submitted.

**Objective:**

You will receive 25 article titles. For each title, determine if it is eligible based on the criteria below. Respond with only one word: "eligible" or "ineligible," as a numbered list.

**Eligibility Criteria:**

An article is considered eligible if it meets both of the following criteria:

1. The study is a randomized controlled trial (RCT). Acceptable terms include:

- Randomised controlled trial, Randomized controlled study, Randomized trial, Randomized study, Randomized control trial, Randomized control study, Randomized clinical trial, Randomized clinical study.

- Terms that describe specific types of RCT designs are also acceptable when used with RCTs: Parallel, parallel-group, non-inferiority, crossover, cross-over, factorial, pragmatic.

2. The study involves the use of digital health technology (DHT) for cognitive behavioral therapy (CBT). DHT must involve computing platforms, connectivity, software, or sensors used in healthcare.

**Exclusion Criteria:**

An article is ineligible if it meets any of the following conditions:

- The title includes terms like: "Proof of concept," "Feasibility," "Preliminary efficacy," "Pilot study," "Protocol," "First results," "Preliminary results," "Design," "Economic evaluation," "Health-economic evaluation," "Secondary analysis," "Qualitative study," "Follow-up," "Longitudinal study," "Short term findings," "Methods," "Letter," "Erratum," "Commentary," "Insights," "Lessons learned," "Reflections," "Correlation study," "Quasi," "Survey," "Process economic evaluation," "Bibliometric analysis," "Meta-analysis," "Systematic review," "Scoping review."

- The study does not explicitly state it is an RCT and does not involve DHT for CBT.

- The title is not written in English.

**Examples of Eligible Articles:**

1. "Internet-delivered cognitive therapy for social anxiety disorder in Hong Kong: A randomized controlled trial."

Response: Eligible

2. "Effect of Digital Cognitive Behavioral Therapy for Insomnia on Health, Psychological Well-being, and Sleep-Related Quality of Life: A Randomized Clinical Trial."

Response: Eligible

3. "Randomized Controlled Trial of Nurse-Delivered Cognitive-Behavioral Therapy Versus Supportive Psychotherapy Telehealth Interventions for Chronic Back Pain."

Response: Eligible

**Examples of Ineligible Articles:**

1. "Feasibility and preliminary efficacy of guided internet-delivered cognitive behavioral therapy for insomnia after the loss of a child to cancer: Randomized controlled trial."

Response: Ineligible (Contains "Feasibility" and "Preliminary efficacy")

2. "CBT-based Online Self-help Training to Reduce Fear and Distress After Cancer (CAREST Randomized Trial): 24 Months Follow-up Using Latent Growth Models and Latent Class Analysis."

Response: Ineligible (Contains "Follow-up")

3. "Impact of online Mindfulness-Based Cognitive Therapy on suicidal ideation: A secondary analysis of a randomized trial of Mindful Mood Balance."

Response: Ineligible (Contains "Secondary analysis")

Now, screen the following titles using the provided eligibility and exclusion criteria and respond with a numbered list selecting only "eligible" or "ineligible" for each title:

Box B. Abstract Information Extraction Standardized Prompt.

Please extract the following data from the given abstract and present it in a CSV-compatible format. Each value should be separated by a comma, and the columns should be named as follows:

- **Digital Health Technology Used**

- **WHO User Category** (options:

Person- Persons are members of the public who are potential or current users of health services including patients,

Provider- Health care providers or providers in training such as medical students are members of the health workforce who deliver health interventions,

Data Services- Activities related to data management, use, and data governance compliance,

Health Management- Health management and support personnel are involved in the administration and oversight of health systems)

- **Purpose** (options:

Education- Providing learning,

Treatment- The treatment arm in the trial design,

Participant- Focused directly on participant engaging, screening, recruitment, and retention,

Assistance- Helping to facilitate the clinical trial as a complement to treatment- typically an administrative component, not a treatment)

- **Health Specialty** (options: American Medical Association recognized specialties)

- **Trial Registry Number**

If the data element belongs to more than one category, list all that apply using spaces to separate multiple categories instead of commas. For any data not reported in the abstract, return "not reported". Here's the CSV format to use:

DHT Used,DHT User,DHT Purpose,Health Specialty,Trial Number

<Extracted Data>,<Extracted Data>,<Extracted Data>,<Extracted Data>,<Extracted Data>

Box C1. Methods Region Sectionalization for Participant Eligibility Standardized Prompt.

You are given the methods section of a digital health technology-enabled randomized controlled trial (RCT). Your task is to extract the exact text that specifies the inclusion and exclusion criteria for participant eligibility. The inclusion criteria outline the conditions participants must meet to be included in the study, while the exclusion criteria specify conditions that disqualify participants from the study. Return the identified participant eligibility criteria text exactly as it is written, without making inferences, summaries, or modifications.

Box C2. Methods Region Participant Eligibility Standardized Prompt.

The input file contains the participant eligibility criteria from the methods section of a digital health technology enabled randomized controlled trial (RCT) manuscript.

Your task is to identify any participant inclusion or exclusion criteria related specifically to the use of the digital health technology.

For each file:

1. Extract any **inclusion criteria** directly relating to access, usage, or requirements for the digital health technology.

2. Extract any **exclusion criteria** directly relating to the inability to access, use, or meet requirements for the digital health technology.

3. If no inclusion or exclusion criteria are reported relating to the digital health technology, respond with "not reported" for both categories.

The output should be formatted as a CSV-compatible structure with the following format:

Inclusion Criteria,Exclusion Criteria

"<list inclusion criteria here>","<list exclusion criteria here>"

If multiple criteria exist, list them in the same field separated by semicolons. For example:

Inclusion Criteria,Exclusion Criteria

"Criteria 1; Criteria 2","Criteria 1; Criteria 2"

Box D1. Results Region Participant Baseline Data Table Standardized Prompt for PDFs.

"Task 1: The input file is from a published randomized controlled trial. Does the file contain a table in true row and column format? If the file contains a true table respond yes. If there is not a true table, respond no.

Task 2: Does the table contain baseline participant demographics and characteristics data? If the file contains a baseline participant demographics table, respond with yes. If the table does not contain baseline demographic details, respond with no. Only evaluate tables for baseline demographic details, not summaries or descriptions of such tables.

Task 3: Conduct a binary evaluation of the reported table variables Age, Sex/Gender, Race/Ethnicity, Education, Income, Employment Status, Housing Status, Disability Status, Insurance Status, Internet Access, Environmental Safety. Write 1 if the variable is reported or 0 if the variable is not reported. The final response should consist of only the following: Task 1 yes or no; Task 2 yes or no; and Task 3 the 12 binary reporting scores."

Box D2. Results Region Participant Baseline Data Table Standardized Prompt for XMLs.

Read the provided XML file for a randomized controlled trial.

Locate the baseline participant demographics/characteristics table in the results section.

If the table is not found, return 'Table Not Reported' and do not proceed with analysis.

If the table is found, evaluate the presence of the following variables: Age, Sex, Race, Education, Income, Employment Status, Housing Status, Disability Status, Insurance Status, Internet Access, Gender, Environmental Safety.

For each variable, return 1 if it is reported in the table, otherwise return 0.

Output the results in the following JSON format:

Table_reported:

variables

Age:

Sex/Gender:

Race/Ethnicity:

Education:

Income:

Employment Status:

Housing Status:

Disability Status:

Insurance Status:

Internet Access:

Environmental Safety:
