# Supplementary material for: Retrieval-to-abstraction LLM-assisted literature synthesis: Case demonstrations in digital health technology-enabled randomized controlled trial outcome reporting
Source: PLOS Digit Health. 2026 Oct 9;5(10):e0001768. doi: 10.1371/journal.pdig.0001768 (PMC13652274; doi:10.1371/journal.pdig.0001768)
Supplement: Supplementary file 2 [file pdig.0001768.s002.docx]

**SUPPLEMENTAL NOTE 2: TITLE SCREENING ELIGIBILITY CRITERIA**

Title Screening: Inclusion criteria require an element from both RCT and DHT to be satisfied.

1.1 Randomized Controlled Trial (RCT)
    1.1.1 Other acceptable synonyms:
          1.1.1.1 Randomised controlled trial
          1.1.1.2 Randomized controlled study or randomised controlled study
          1.1.1.3 Randomized trial or randomised trial
          1.1.1.4 Randomized study or randomised study
          1.1.1.5 Randomized control trial or randomised control trial
          1.1.1.6 Randomized control study or randomised control study
          1.1.1.7 Randomized clinical trial or randomised clinical trial
          1.1.1.8 Randomized clinical study or randomised clinical study
          1.1.1.9 Parallel, parallel-group, parallel-cohort
          1.1.1.10 Crossover or cross-over
          1.1.1.11 Factorial
          1.1.1.12 Pragmatic
          1.1.1.13 Prospective

2.1 Digital Health Technology (DHT)

2.1.1 Examples of broad terms (specific terms are also eligible)
      2.1.1.1 Mobile health, mobile apps, telehealth, telemedicine
      2.1.1.2 Text message communication, social media
      2.1.1.3 Software as a medical device
      2.1.1.4 Artificial intelligence and machine learning
      2.1.1.5 Wearable or wireless medical devices
      2.1.1.6 Data management, cloud, cybersecurity, interoperability
      2.1.1.7 Virtual reality

3.1 Exclusion Criteria

3.1.1	Not a randomized controlled trial

3.1.2	No digital health technology (exclude DHT homonyms e.g., “remote” meaning rural or “tablet” meaning pill)

3.1.3	Not written in English

3.1.4	Not a randomized controlled trial result, outcome, or final finding (exclude: protocol, pilot, feasibility, methods, design, letter, erratum, commentary, lessons learned, reflections, correlation study, quasi, survey, proof-of-concept, process evaluation, secondary analysis, nested economic evaluation, short term findings, qualitative study, of follow up such as one-year or five-year)

3.1.5	More than one clinical trial, such as a comparison of two trials

3.1.6	A review (meta-analysis, bibliometric analysis, systematic or scoping review)
