# Supplementary material for: Retrieval-to-abstraction LLM-assisted literature synthesis: Case demonstrations in digital health technology-enabled randomized controlled trial outcome reporting
Source: PLOS Digit Health. 2026 Oct 9;5(10):e0001768. doi: 10.1371/journal.pdig.0001768 (PMC13652274; doi:10.1371/journal.pdig.0001768)
Supplement: Supplementary file 3 [file pdig.0001768.s003.docx]

**SUPPLEMENTAL NOTE 3: ABSTRACT ANNOTATION GUIDELINES**

**Annotation Guideline: Abstract**

***Randomizes Controlled Trials***

Purpose: The purpose of this task is to understand reporting patterns of digital health technology elements in published randomized controlled trials. We have identified 5 primary categories of interest contained in the manuscript abstract section:

1. Name of the digital health technology
2. User of the digital health technology
3. Purpose of the digital health technology
4. Health Specialty of the clinical trial
5. Clinical Trial Number or registry number

Annotation example MedTator 1.3.16 (<https://medtator.ohnlp.org/>)

MedTator

I. Open the MedTator webpage.
II. Drag the schema into the upper left corner that says “Drop a Schema File Here”.

III. Drag and drop the txt files in the left side.

**Abstract Annotation Guidelines**

Annotate the most descriptive occurrence of each of the following five elements (if necessary to capture relevant detail, it is acceptable to add more than one annotation for an attribute). Most annotations will take place in the first and last sentences.

I. **DHT Name**: Type or specific name of the digital health technology in the trial.
   a. Attribute
      i. General: This is a general term used for the digital health technology
         1. Example: VR, immersive 3D VR, and smartphone
      ii. Specific: This is a product or company name for the technology
          1. Example: Wii, Oculus, and Kinect
II. **DHT User**: Who is using the digital health technology?

Based on the World Health Organization Categorization of DHT Users [*https://iris.who.int/bitstream/handle/10665/373581/9789240081949eng.pdf?sequence=1*](https://iris.who.int/bitstream/handle/10665/373581/9789240081949eng.pdf?sequence=1)

a. Attribute
   i. Person: Persons are members of the public who are potential or current users of health services
      1. Example: patients, clients, and health service users
   ii. Provider: Health care providers are members of the health workforce who deliver health interventions
       1. Example: doctor, nurse, and physician assistant
   iii. Management: Health management and support personnel are involved in the administration and oversight of health systems. Interventions within this category reflect managerial functions related to supply chain management, health system financial management, human resource management
        1. Example: hospital administrator, and admin supervisor
   iv. Data: This consists of crosscutting functionality to support a wide range of activities related to data management, use, and data governance compliance
       1. Example: data storage

III. **DHT Purpose**: What is the digital health technology being used for?
     a. Attribute
        i. Education: DHT used to provide learning
           1. Example: online course about disease, surgical training
        ii. Assistance: DHT used to help facilitate, or add to, the treatment but not the treatment itself
            1. Example: distraction tool, operating room tour
        iii. Patient: DHT relating to screening, recruitment, or retention
             1. Example: participant consent
        iv. Treatment: The clinical trial treatment
            1. Example: physical rehabilitation, cognitive behavioral therapy
IV. **Health Specialty**: Field of health care or specific medical condition studied
    a. Attribute
       i. Specialty: American Medical Association recognized specialties
          1. Oncology, pediatrics, neurology
       ii. Condition: Specific disease, condition, or treatment
           1. Anxiety, fear of public speaking, post traumatic stress disorder
V. **Trial Number**: Clinic trial or registry number
   a. Example: ClinicalTrials.gov *NCT04093154*

**Full Examples: DHT_Name**; **DHT_User**; **DHT_Purpose**; **Health Specialty**, **Trial Number**

EXAMPLE 1: BACKGROUND: Asymmetric movements with both hands contributed to the improvement of spatially coupled motion. Thus, the aim of this study was to investigate the effects of an asymmetric training program using **virtual reality** reflection equipment on **upper limb function** in **stroke patients**. METHODS: Twenty-four stroke patients were randomly allocated to an experimental group (n=12) or a control group (n=12). Both groups participated in conventional **physical therapy** for 2x30 min/d, 5 d/wk, for 4 weeks. The experimental group also participated in an asymmetric training program using virtual reality reflection equipment, and the control group participated in a symmetric training program. Both asymmetric and symmetric programs were conducted for 30 min/d, 5 d/wk, for 4 weeks. To compare upper limb function before and after intervention, the Fugl-Meyer Assessment (FMA), the Box and Block Test (BBT), grip strength, range of motion (ROM), and spasticity were assessed. RESULTS: Both groups showed significant increases in upper limb function, excepting spasticity, after intervention (P<.05, 1-way repeated-measures analysis of variance ANOVA). A significant group-time interaction was demonstrated only for shoulder/elbow/wrist items of FMA, BBT, grip strength, and ROM of wrist flexion, extension, and ulnar deviation (P<.05, 2-way repeated-measures ANOVA). CONCLUSIONS: This study confirms that the asymmetric training program using virtual reality reflection equipment is an effective intervention method for improving upper limb function in stroke patients. We consider that an additional study based on a program using virtual reflection, which is more functional than performing simple tasks, and consisting of tasks relevant to the activities of daily living be conducted.

EXAMPLE 2: Aim: The aim of this study was to examine the effect of a water-friendly Projector-Based Hybrid **Virtual Reality (VR)** dome environment combined with standard pharmacological treatment on pain in **young children undergoing burn wound care** in **hydrotherapy**. Methods: This study was a prospective, within-subject crossover trial of 38 **children aged 6 months to 7 years old** (mean age =1.8 years old). Each hydrotherapy procedure was divided into two equivalent wound care segments (No hybrid VR during one segment vs. Hybrid VR during the other segment, treatment order was randomized). Pain was measured using the 0-10 FLACC (Face, Legs, Activity, Cry Consolability scale) and the 0-10 NRS-obs (Numerical Rating Scale-obs). Results: Projector-Based Hybrid VR significantly reduced procedural pain levels measured by the FLACC (p= 0.026) and significantly increased patients' comfort levels (p= 0.002). Patients' pain levels rated by the nurses using the NRS-obs were non-significant between both groups (p=0.135). No side effects were reported. Conclusion: Projector-Based Hybrid VR helped in **reducing the pain related to hydrotherapy procedures** in young children with burn wound injuries. This is the first study using virtual reality **distraction** with young children, and our findings are especially important because a large percentage of pediatric burn patients are very young. Additional research and development are recommended. (C) 2020 Elsevier Ltd and ISBI. All rights reserved. Trial registration: **NCT04166747**.
