# Supplementary material for: Retrieval-to-abstraction LLM-assisted literature synthesis: Case demonstrations in digital health technology-enabled randomized controlled trial outcome reporting
Source: PLOS Digit Health. 2026 Oct 9;5(10):e0001768. doi: 10.1371/journal.pdig.0001768 (PMC13652274; doi:10.1371/journal.pdig.0001768)
Supplement: Supplementary file 4 [file pdig.0001768.s004.docx]

**SUPPLEMENTAL NOTE 4: PARTICIPANT ELIGIBILITY ANNOTATION GUIDELINES**

**Annotation Guideline: Participant Eligibility**

***Randomizes Controlled Trials***

Purpose: The purpose of this task is to understand reporting patterns of digital health technology related participant eligibility in published randomized controlled trials. Annotate all occurrences of the following elements, only if they relate to the DHT:

1. Internet access
2. Device access
3. Account access
4. Technology knowledge
5. Disability
6. Medical condition
7. Language
8. Other

These elements will be annotated using MedTator: <https://medtator.ohnlp.org/?st=yes>

**Annotation Guideline: Participant Eligibility**

1. Internet access: Participant provided internet access.

Example:

The inclusion criteria for this study:

• participants must have **internet access**,

Annotation:

**Internet access**: Inclusion, Present

2. Device access: Participant provided access to the trial DHT (e.g. iPad)

Example:

The inclusion criteria for this study:

To be have **an Android system** on **the mobile phones**

Annotation:

**An Android system**: Inclusion, Present

**The mobile phones**: Inclusion, Present

3. Account access: Participant provided account (e.g. social media account)

Example:

**Inclusion criteria:**

Finally, participants needed to have access to a computer, a cellphone, **email**, and the Internet

Annotation:

**Email**: Inclusion, present

4. Technology knowledge: Participant can operate trial DHT (e.g. send text message)

Example:

**Inclusion criteria:**

To be able to complete the registration, the participants needed to be **computer literate enough to access and navigate the website** via a computer, tablet, or smartphone."

Annotation:

**Computer literate enough to access and navigate the website**: Inclusion, present

5. Disability: DHT-related disability (e.g. blindness for a VR trial)

Example:

**Inclusion criteria:**

- h) not having any **mental** and **communication problems** and **visual impairments**."

Annotation:

**Mental** **impairments**: Inclusion, absent

**Communication problems**: Inclusion, absent

**Visual impairments**: Inclusion, absent

6. Medical condition: DHT-related medical condition (e.g. vertigo for a VR trial)

Example:

Exclusion criteria: Had medical contraindications for a VR set (such as **neurologic**, **ophthalmologic**, or **otolaryngologic disorders**)

Annotation:

**Neurologic disorders**: Exclusion, present

**Ophthalmologic disorders**: Exclusion, present

**otolaryngologic disorders**: Exclusion, present

7. Language: Trial language

Example:

Participants were recruited under the following inclusion criteria: able to understand **Mandarin Chinese**;

Annotation:

**Mandarin Chinese**: Inclusion, Present

8. Other

Example:

**Exclusion Criteria:**

- "The children were excluded from the study when they **wanted to take off the virtual headset during the procedure**."

Annotation:

**Wanted to take off the virtual headset during the procedure**: Exclusion, Present

Each element has two attributes:

1. Eligibility
   a. Inclusion: Content listed in the inclusion criteria
   b. Exclusion: Content listed in the exclusion criteria
2. Assertion
   a. Present
   b. Absent
   c. Other

**Full Examples: Internet Access**, **Device Access**, **Account Access**, **Technology Knowledge,** **Disability**, **Medical Condition**, **Language**, **Other**

EXAMPLE 1:

The inclusion criteria for this study:

• To be enrolled in Fundamentals of Nursing II for the first time,

• To be have **an Android system** on **the mobile phones**,

• To be have **internet access**,

• To be have no previous experience in tracheostomy care.

The exclusion criteria for this study:

• To be having attended at least one of the theoretical classes, demonstrations, and small group studies,

• To be not having taken the first or last skill performance assessments.

EXAMPLE 2:

**Inclusion criteria:**

"Inclusion criteria comprised patients aged more than 18 years who had first experience with EGD and indication for unsedated EGD with 2% lidocaine spray 5 mL for pharyngeal anesthesia."

**Exclusion criteria:**

"The patients were excluded if they received a diagnosis of **vertigo**, **epilepsy**, **eye** and **ear problems**, **claustrophobia**, **achluophobia**, or **previous neurosurgery**. Patients with **active nausea**, **vomiting**, or **unstable hemodynamic conditions** and who were **indisposed to wear three-dimension (3D) VR** were excluded."

Note: “**eye** and **ear problems**” is annotated by highlighting eye, then hold the command button and highlight problems.
